# Relationship between Periodontitis and Pre-Eclampsia: A Meta-Analysis

**DOI:** 10.1371/journal.pone.0071387

**Published:** 2013-08-19

**Authors:** Fabrizio Sgolastra, Ambra Petrucci, Marco Severino, Roberto Gatto, Annalisa Monaco

**Affiliations:** Department of Life, Health and Environmental Sciences, University of L'Aquila, L'Aquila, Italy; National Cancer Center, Japan

## Abstract

**Background:**

Studies have suggested controversial results regarding a possible association between pre-eclampsia (PE) and periodontal disease (PD) and no meta-analysis has been performed to clarify this issue.

**Methods:**

A literature search of electronic databases was performed for articles published through March 24, 2013, followed by a manual search of several dental and medical journals. The meta-analysis was conducted according to the recommendations of the Cochrane Collaboration and PRISMA. Odds ratios (ORs) and 95% confidence intervals (CIs) were calculated. Heterogeneity was assessed with the χ^2^-based Cochran Q test and I^2^ statistic. The level of significance was set at *P* **<**0.05.

**Results:**

Fifteen studies were included, including three cohort and 12 case-control studies. A positive association was found between PE and PD (OR 2.17, 95% CI 1.38–3.41, *P* = 0.0008). However, a high and significant heterogeneity was found (χ^2^ = 62.42, *P*<0.00001, I^2^ = 75%). In most cases, subgroup analysis had low power to detect significant differences between PE and non-PE groups.

**Conclusion:**

Based on the findings of the meta-analysis, PD appears to be a possible risk factor for PE. However, given the important differences in the definitions and diagnoses of PD and PE among the studies, as well as their lack of good methodological quality, future trials are needed to confirm the results of the present meta-analysis.

## Introduction

Infection and inflammation continue to be at the forefront of etiologic theories as causative factors of adverse pregnancy outcomes, such as stillbirth and growth restriction, that affect many women each year. Previous studies have demonstrated a link between infection or inflammation and preterm birth, preeclampsia (PE), and other adverse outcomes thought to be secondary to poor placentation [1]–[4]. The prevalence of PE, a multisystem disorder of unclear etiology that is exclusive to human pregnancy, ranges from 2% to 7% in developed countries. PE results in high maternal and neonatal morbidity and mortality rates, attributable to complications affecting different organs and systems. In emerging countries, the prevalence of PE is more than 10% [4], and the condition is the main cause of maternal death [5].

PE occurs usually after 20 weeks of gestation. It is characterized by an abnormal vascular response to placentation, manifesting as generalized vasospasm, activation of the coagulation system, and reduced organ perfusion affecting the kidney, liver, and brain [4]. Two syndromes are included in the definition of PE: maternal, characterized by endothelial cell activation, perturbations in volume and blood pressure control, gradual maternal blood pressure elevation, proteinuria, and generalized edema; and fetal, manifested primarily by intrauterine growth restriction [6]–[8]. Putative PE risk factors include advanced maternal age, multifetal pregnancies, maternal prepregnancy obesity, pregestational hypertension, renal disorders, and diabetes mellitus [9]–[12]. In recent years, infection has been reported to be important in the pathogenesis of PE, both in terms of its initiation and its potentiation [7], [8], [13].

Several studies have suggested that periodontal disease (PD), a chronic inflammatory oral infection, may be associated with an increased risk for PE development [14]–[19]. PD affects 20% to 50% of pregnant women, especially economically disadvantaged women [20], [21]. In this inflammatory pathology, the dental plaque – which is a biofilm predominated by Gram-negative anaerobic microorganisms – destroys the tooth-supporting tissues. Oral microorganisms initiate PD, but the periodontal breakdown is primarily mediated by the host inflammatory response [22], [23]. PD may burden pregnant women systemically with endotoxins, inflammatory cytokines, and oxidative stressors at the maternal-fetal interface [17]. Thus, PD may be a vascular stressor that plays a role in the development of PE in pregnant women.

Contradictory findings exist regarding the relationship between PD and PE [15], [24]–[26], and a previous systematic review did not clarify this possible association [27]. Therefore, there is a need for a systemic assessment of the literature on the possible association between PD and PE. The aim of the present systematic review and meta-analysis was to assess the scientific evidence on the possible association between PD and PE.

## Materials and Methods

The present meta-analysis was conducted according to the Preferred Reporting Items for Systematic Reviews and Meta-analysis [28] (PRISMA) guidelines.

### Search

The following databases were searched from their earliest records through March 24, 2013: MEDLINE, Cochrane Controlled Clinical Trial Register, Cochrane Database of Systematic Reviews, Database of Abstracts of Reviews of Effects, CINAHL, Science Direct, ISI Web of Knowledge, and SCOPUS. The following search algorithm was used to explore databases, by using Boolean operators and the asterisk symbol (*) as truncation: (“Periodontitis”[Mesh] OR “Chronic Periodontitis”[Mesh] OR “Periodontal Diseases”[Mesh] OR “Periodontal Pocket”[Mesh] OR “Periodontal Attachment Loss”[Mesh] OR “Tooth Mobility”[Mesh] OR periodontitis OR periodontal disease* OR periodontal pocket* OR attachment loss OR alveolar bone loss OR pocket depth OR clinical attachment level) AND (“Pre-Eclampsia”[Mesh] OR “Eclampsia”[Mesh] OR “Hypertension, Pregnancy-Induced”[Mesh] OR preeclampsia OR pre-eclampsia OR eclampsia OR gestosis OR pregnancy hypertension OR pregnancy hypertensive). In the CINAHL, SCOPUS, ISI Web of Knowledge, and Science Direct databases, the MeSH terms were not used.

In addition, a manual search was performed of issues of the last 15 years of the following journals: *Journal of Periodontology, Journal of Clinical Periodontology, Journal of Dental Research, Journal of Periodontal Research, Periodontology 2000, Journal of Dentistry, Journal of the American Dental Association, Journal of Clinical Dentistry, Clinical Oral Investigations, Acta Obstetricia and Gynecologica, Journal of Obstetrics and Gynaecology, British Journal of Obstetrics and Gynecology, American Journal of Obstetrics and Gynecology,* and *Obstetrics and Gynecology*. To be as inclusive as possible, no restrictions were applied with regard to the publication year of the studies or to language. The references of all selected full-text articles and related reviews were scanned.

### Study Selection

Screening was performed independently by two blinded reviewers (FS and MS). Interreviewer reliability in the study selection process was determined by the Cohen *κ* test, assuming an acceptable threshold value of 0.61 [29], [30]. In case of disagreement on the inclusion or the exclusion of studies, this issue was discussed until consensus was reached by the reviewers who selected the studies (FS and MS).

### Eligibility Criteria

The study selection process was performed by two blinded reviewers (RG and AM) in two phases. In the first phase, the studies were analyzed according to the following inclusion criteria (A): 1) cross-sectional, prospective cohort or case-control studies, 2) studies analyzing the association between PD and PE, 3) PD defined by clinical or radiographic parameters, 4) studies reporting clear definition of PD and PE, and 5) studies conducted on adult human subjects (age >18 years). Only studies that met all inclusion criteria in (A) were admitted to the second phase, which consisted of the analysis of the preselected studies according to the following exclusion criteria (B): 1) studies including patients with systemic disease, 2) studies that did not report adjustment for known confounder factors, 3) studies not reporting adequate data, 4) ancillary or duplicate studies, and 5) no outcome of interest.

### Data Extraction

Data were collected by two independent reviewers (FS and RG). The following data were extracted from the included studies: year of publication, country, study design, demographic characteristics of participants, definition of PE and PD, and main findings. If data were presented both numerically (in tables or text) and graphically (in figures), only numeric data were considered for extraction. The reviewers cross-checked all extracted data. Disagreements were resolved by discussion until consensus was reached.

### Risk of Bias

Assessment of risk of bias was performed according to the Newcastle-Ottawa Scale by two independent reviewers (FS and AP). The level of agreement between reviewers was 0.76.

### Quantitative Analysis

#### Measure of effect size

Data were combined for meta-analysis with a statistical software (RevMan, Version 5, 2008, The Nordic Cochrane Center, The Cochrane Collaboration, Copenhagen, Denmark). For dichotomous data, the odds ratio (OR) and 95% confidence interval (CI) were calculated. Due to the expected interstudy heterogeneity, a random effect model was used. The pooled effect was considered significant if *P* was **<**0.05. Forest plots for each meta-analysis present the raw data, OR (displayed as blocks), and CIs (displayed as lines) for the chosen effect, the heterogeneity statistic (I^2^), total number of participants per group, and overall OR in the random effect model.

#### Subgroup analysis

Subgroup analysis was performed according to the type of study (cohort or case-control study), severity of PD (mild, moderate, or severe), definition of PD (defined by probing pocket depth [PPD] and/or clinical attachment level [CAL]) and security of PD diagnosis (defined according to the criteria suggested by Nibali et al. 2013 [31]).

#### Heterogeneity

Heterogeneity was assessed by the χ^2^-based Q-statistic method and I^2^ measurement, with significance indicated by *P*<0.1.

#### Publication bias

The publication bias was investigated by two methods. Visual detection was used to analyze the funnel plots [32]. Quantitative analysis was performed by the regression asymmetry test [33] and the trim-and-fill method [34]. Publication bias was assessed with an additional statistical software (Stata IC version 10.1, StataCorp, College Station, Texas).

## Results

### Search Results

**Figure 1:**
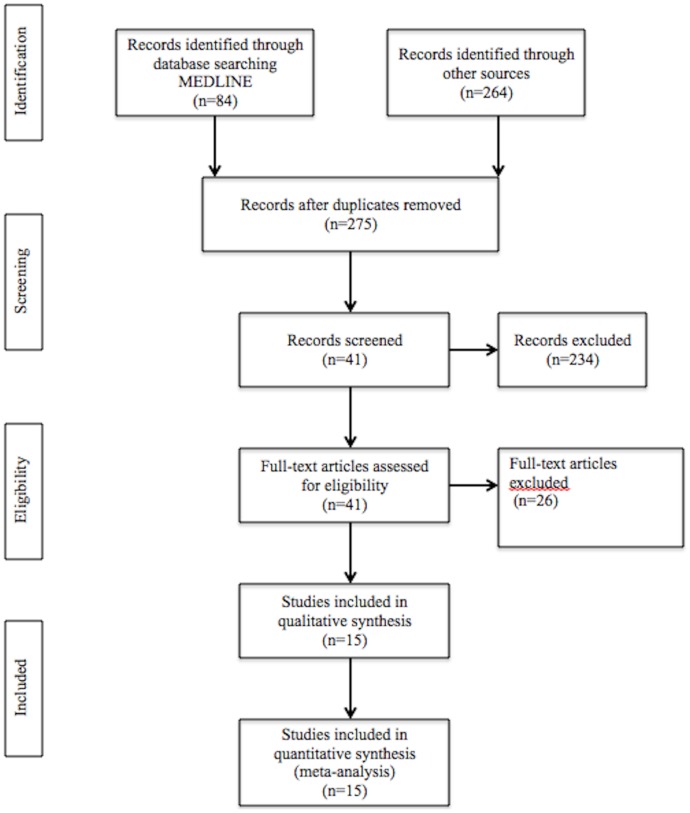
PRISMA Flowchart of the search strategy.

A total of 348 articles were found through the electronic and manual searches. After removing duplicates, 275 articles were found (inter-reviewer agreement, κ = 0.78), including 84 in MEDLINE, 4 in Cochrane Controlled Clinical Trial Register, 69 in CINHAL, 55 in Science Direct, 9 in Scopus, and 55 in ISI Web of Knowledge. Then, 234 papers were excluded on the basis of the evaluation of the title and abstract, leaving 41 articles to be assessed for eligibility (κ = 0.84). Of these, 11 articles were excluded in the first phase of the selection process, for not satisfying one or more inclusion criteria (κ = 1) [19], [35]–[44]. Fifteen of the remaining 30 [45]–[59] articles were further excluded (κ = 1). Finally, 15 studies [14], [15], [19], [24]–[26], [60]–[68] qualified for inclusion in the systematic review and meta-analysis (κ = 1). The list of the excluded studies and their reasons for exclusion are provided in Table 1. A PRISMA flowchart is provided in Figure 1.

**Table 1:** List of excluded studies and reasons for exclusion.

Study	Year of publication	Criteria for exclusion	Type of study
Swati et al. [35]	2012	A.2	Case-control study
Abati et al. [45]	2012	B.3	Case-control study
Boggess et al. [36]	2012	A.3	Cross-sectional study
Cetin et al. [37]	2012	A.1	Review
Hirano et al. [46]	2012	B.5	Case-control study
Piscoya et al. [38]	2012	A.3	Case-control study
Swati et al. [47]	2012	A.2	Case-control study
Lopez-Jaramillo et al. [39]	2011	A.1	Comment
Sayar et al. [48]	2011	B.2	Case-control study
Matevosyan [40]	2011	A.1	Meta-analysis
Kunnen et al. [27]	2010	A.1	Systematic review
Horton et al. [49]	2010	B.4	Cohort study
Nabet et al. [50]	2010	B.5	Case-control
Vergnes [41]	2008	A.1	Systematic review
Ruma et al. [51]	2008	B.4	Cohort study
Conde-Agudelo et al. [42]	2008	A.1	Systematic review
Dasanayake [43]	2007	A.1	Comment
Canacki et al. [52]	2007	B.4	Case-control
Cota et al. [53]	2006	B.4	Case –control
Khader et al. [54]	2006	B.3	Meta-analysis
Castaldi et al. [55]	2006	B.5	Cross-sectional study
Boggess et al. [56]	2006	B.4	Cross-sectional study
Meurman et al. [57]	2006	B.2	Retrospective study
Contreras et al. [58]	2006	B.2	Case-control study
Xiong et al. [21]	2006	A.1	Systematic review
Oettinger-Barak et al. [59]	2005	B.2	Case-control study

### Description of Included Studies

The main characteristics of the included studies are described in Table 2. Three of 15 included studies were cohort studies, whereas the remaining 12 were case-control studies. The definitions of PE and PD varied greatly among the included studies. The sample size varied from 54 patients in the study of Chaparro et al. [60] to 1206 in the study of Siqueira et al. [67]. Three studies [24], [26], [66] did not found a positive association between PD and PE, whereas the remaining studies reported a significant association.

**Table 2:** Characteristics of the included studies.

Author, Year	Design	Country (Race/ethnicity)	Population	Age (y)	Definition of PE	Definition of PD	Findings
Canakci, 2007	C/C	Erzurum, Turkey	20 Mild PE, 18 Severe PE, 21 Control	Mild PE: 24.1±3.9; Severe PE: 23.6±4.2; Control: 24.7±4.5	PE: DBP ≥90 mmHg and PU (300 mg/24h US) and edema; Mild PE: BP ≥140/90 mmHg on ≥2 occasions 6h apart, w/or w/o PU; Severe PE: SBP ≥160 or DBP ≥110 mmHg on 2 occasions ≥6h apart and PU ≥5 g/24h US or ≥3 l on dipstick in ≥2 random clean-catch samples ≥4h apart	BOP and ≥4 mm PPD on 1–15 sites (Mild PD) or ≥15 sites (Severe PD)	There was a significant association between mild to severe PE and severe PD.
Chaparro, 2012	C/C	Santiago, Chile	11 PE, 43 Control	PE: 28.91±6.11; Control: 27.19±7.18	BP >140/90 mmHg and PU (300 mg/24h US)	≥4 teeth w/≥1 sites w/PPD ≥4 mm and CAL ≥3 and BOP	Increased IL-6 levels in GCF (OR = 1.06, *P* = 0.02, CI 95% 1.007–1.117) in early pregnancy were associated with increased PE risk.
Ha, 2011	C/C	Seoul, Korea	16 PE, 48 Control	PE: 32.69±5.30 (21–40); Control: 32.69±4.40 (21–40)	BP >140/90 mmHg on 2 occasions and 1+ or more PU on a random US	CAL ≥3.5 mm on 2–3 sites (Localized PD) or on ≥4 sites (Generalized PD) of different teeth	PE could be associated with maternal PD.
Wang, 2012	C/C	Niigata, Japan (Japanese)	13 PE, 106 Control	-	SBP ≥140 or DBP ≥90 mmHg on 2 occasions and PU (≥300 mg/d) after 20 GW	>60% of sites with CAL ≥3 mm	Polymorphism and subgingival DNA level of *A. actinomycetemcomitans* were significantly associated with PE, independent of PD.
Srinivas, 2009	Co	Philadelphia, PA, USA (AA)	876 Patients	-	>140/90 mmHg with PU	CAL ≥3 mm on ≥3 teeth	No significant association was found between PD and PE.
Siqueira, 2008	C/C	Belo Horizonte (Multiethnic)	164 PE, 1042 Control	-	SBP >140 or DBP >90 mmHg on 2 occasions after 20 GW and 1+ or more PU	≥4 mm and CAL ≥3 mm at the same site in ≥4 teeth	Maternal PD was observed to be a risk factor associated with PE.
Boggess, 2003	Co	NC, USA (Multiethnic)	39 PE, 763 Control	-	SBP >140 or DBP >90 mmHg on 2 occasions and 1+ or more PU	PPD ≥4 mm on 1–15 teeth or BOP+ (Mild PD) PPD ≥4 mm on >15 teeth (Severe PD)	Severe PD at delivery was associated with increased PE risk (adj OR 2.4, 95% CI 1.1–5.3).
Da Silva, 2012	C/C	Recife, Brazil	284 PE, 290 Control	-	SBP ≥140 or DBP ≥90 mmHg and PU ≥300 mg/24h or 2+ PU on dipsticks, developed after 20 GW	≥4 teeth with ≥1 sites with PPD ≥4 mm and AL ≥3 mm in the same site	PD could be a risk factor for PE.
Shetty, 2010	C/C		30 PE, 100 Control	-	SBP ≥140 or DBP ≥90 mmHg on >2 occasions 4h apart and 1+ or more PU by dipstick on random US	CAL ≥3 mm and PPD ≥4 mm	PD at enrollment (OR = 5.78, 95% CI 2.41–13.89) and w/i 48h of delivery (OR = 20.15, 95% CI 4.55–89.29) may be associated with increased PE risk.
Taghzouti, 2012	C/C	Quebec, Canada (Multiethnic)	92 PE, 245 Control	-	SBP ≥140 or DBP ≥90 mmHg and 1+ or more PU	≥4 sites with PPD ≥5 mm and CAL ≥3 mm at the same sites	PD was not associated with PE (adj OR = 1.13, 95% CI = 0.59 to 2.17).
Kumar, 2012	Co	New Delhi	35 PE, 305 Control	PE: 22.32±2.75; Control: 22.32±2.79	SBP ≥140 or DBP ≥90 mmHg and PU ≥300 mg/24h or 2+ PU on dipsticks, developed after 20 GW	CAL and PPD ≥4 mm in ≥1 sites	PD was significantly associated with PE.
Canakci, 2004	C/C	Erzurum, Turkey (Turkish)	41 PE, 41 Control	PE: 25.9±5.9; Control: 25.8±5.8	SBP ≥140 or DBP ≥90 mmHg and PU ≥300 mg/24h or 2+ PU on dipsticks, on 2 occasions ≥6h apart if 24h US is unavailable	≥ 4 teeth with ≥1 sites with PPD ≥4 mm and BOP+ and CAL ≥3 mm at the same site	No. of sites with PD ≥4 mm and CAL ≥3 mm was higher among PE patients than among controls (*P<*0.01).
Lohsoonthorn, 2009	C/C	Bangkok, Thailand (Thai)	150 PE, 150 Control	-	SBP ≥140 or DBP ≥90 mmHg and PU ≥30 mg/dl (or 1+ on a urine dipstick) on ≥2 random specimens collected ≥4h apart.	≥1 teeth (Mild PD) or ≥2 nonadjacent teeth (Moderate or Severe PD) with interproximal sites showing PPD ≥4 mm and CAL ≥4 mm (Mild or Moderate PD) or ≥5 mm (Severe PD)	Severe clinical PD was not associated with an increased risk of PE (adj OR = 0.92, 95% CI: 0.26–3.28).
Kunnen, 2007	C/C	Groningen, The Netherlands (Caucasian)	17 PE, 35 Control	PE: 29.5±5.1; Control: 31.7±4.2	DBP ≥90 mmHg on 2 occasions and PU ≥30 mg/dl (or 1+ on a urine dipstick) on ≥2 random specimens collected ≥4h apart.	BOP and PPD ≥4 mm on 1 – 15 sites (Mild PD) or >15 sites (Severe PE)	Severe PD was found in 82% of the PE patients and 37% of the control group (*P* = 0.009).
Politano, 2011	C/C	São Paulo, Brazil	58 PE, 58 Control	PE: 28.62±6.93; Control: 24.69±5.37	SBP ≥140 or DBP ≥90 mmHg after 20 GW and PU ≥300 mg	≥2 sites w/PPD ≥4 mm and CAL ≥4 mm and BOP	There was an association between PD and PE (adj OR 3.73, 95% CI 1.32–10.58).

Legend: C/C, case-control study; Co, cohort study; AA, African-American; GW, gestational weeks; adj, adjusted; US, urine specimen; PD, periodontal disease; PE, pre-eclampsia; PU, proteinuria; BP, blood pressure; SBP, systolic blood pressure; DBP, diastolic blood pressure; GCF, gingival crevicular fluid.

### Quality Analysis

None of the included studies reached the maximum score of the Newcastle Ottawa Scale (Table 3). Only two studies [63], [64] gained the maximum score in the Selection outcome; nine studies [15], [19], [24], [25], [26], [52], [63], [64], [65] had the maximum score in the Comparability outcome; and all studies had a partial score in the Exposure outcome.

**Table 3:** Risk of bias in included studies.

Study	Selection (Max 4 *)	Comparability (Max 2 *)	Exposure (Max 3 *)
Boggess et al., 2003	***	*	**
Canakci et al., 2004	***	**	**
Canakci et al., 2007	***	**	**
Chaparro et al., 2012	***	*	**
Ha et al., 2011	****	**	**
Kumar et al., 2012	***	*	**
Kunnen et al., 2007	***	**	**
Lohsoonthorn et al., 2009	***	**	**
Politano et al., 2011	***	**	**
Shetty et al., 2010	***	**	**
Siqueira et al., 2008	***	*	**
Moura da Silva et al., 2012	****	**	**
Srinivas et al., 2009	***	*	**
Taghzouti et al., 2012	***	**	**
Wang et al., 2012	***	*	**

### Results of the Meta-analysis

**Figure 2:**
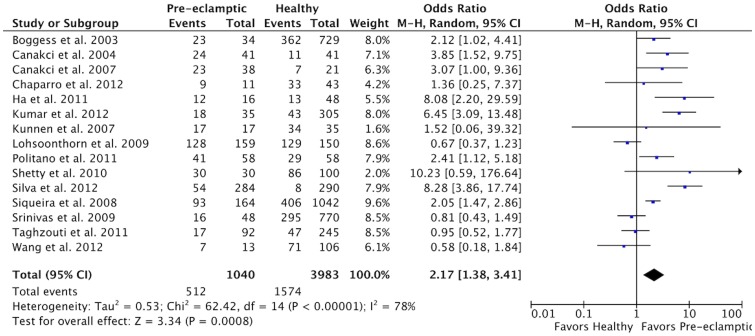
Forest plot for the association between PD and PE.

The results of the meta-analysis showed that an increased risk for PE was present for patients with PD (OR 2.17, 95% CI 1.38–3.41, *P* = 0.0008; Figure 2); however, a high and significant heterogeneity was found (χ^2^ = 62.42, *P*<0.00001; I^2^ = 75%).

#### Subgroup analysis

**Figure 3:**
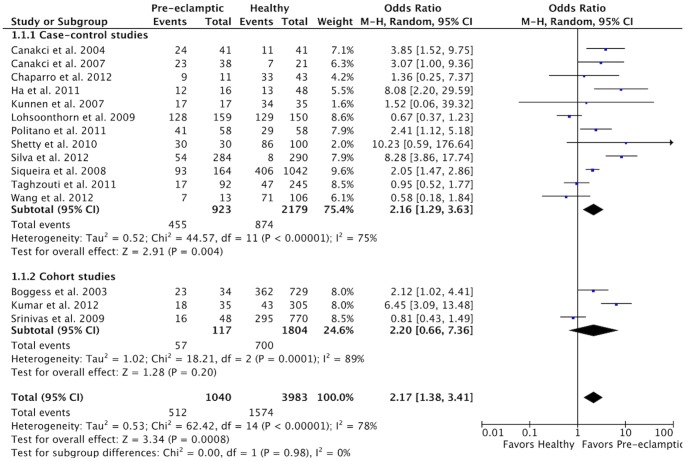
Forest plot for the subgroup analysis according to the type of study design.

An analysis of the results according to study type (Figure 3) revealed an increased risk of PE in PD patients in the case-control (OR 2.16, 95% CI 1.29–3.63, *P* = 0.004) and in the cohort studies (OR 2.20; 95% CI 0.66–7.36, *P* = 0.20). However, this increased risk only remained significant in the case-control subgroup. Heterogeneity was significant in both subgroups (χ^2^ = 44.57, *P*<0.00001, I^2^ = 75% for case-control, and χ^2^ = 18.21, *P* = 0.0001, I^2^ = 89% for cohort studies).

**Figure 4:**
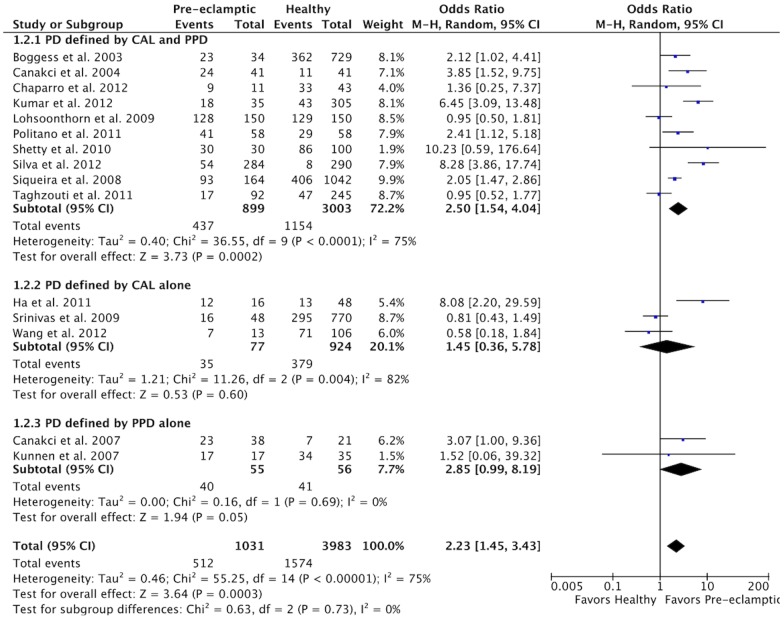
Forest plot for the subgroup analysis according to the PD definition.

When the meta-analysis results were analyzed according to the definition of PD, an increased risk of PE was observed in all subgroups (Figure 4). However, it was significant only in the subgroup in which PD was defined by PPD and CAL (OR 2.50, 95% CI 1.54–4.04, *P* = 0.0002). Heterogeneity was high and significant for the subgroups in which PD was defined by PPD and CAL (χ^2^ = 36.55, *P*<0.0001, I^2^ = 75%) and by CAL alone (χ^2^ = 11.26, *P* = 0.004; I^2^ = 82%), but not for those in which PD was defined by PPD alone (χ^2^ = 0.16, *P* = 0.69; I^2^ = 0%).

**Figure 5:**
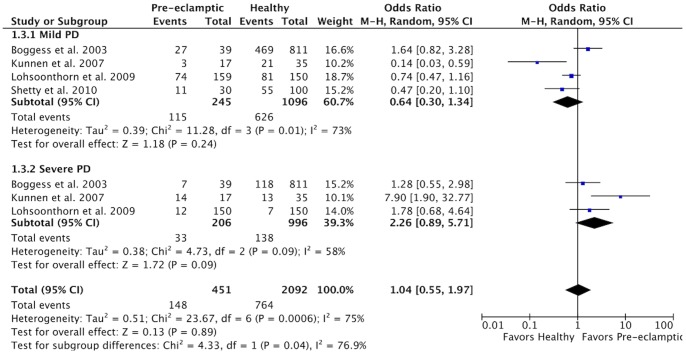
Forest plot for the subgroup analysis according to the PD severity.

**Figure 6:**
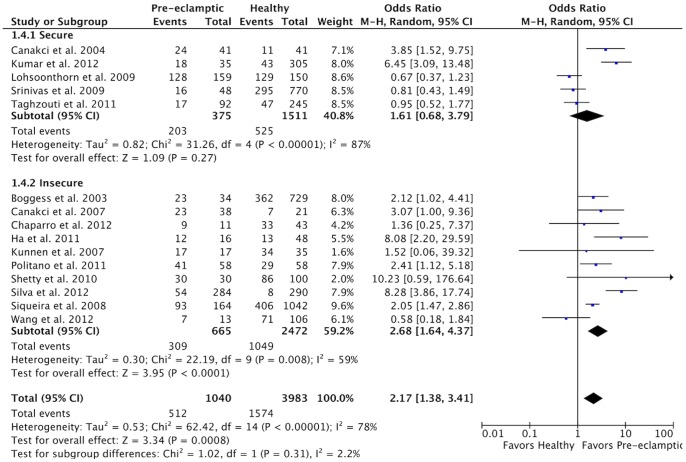
Forest plot for the subgroup analysis according to the PD diagnosis.

When the results were analyzed according to PD severity, no significant risk was observed for mild PD or for severe PD (Figure 5). Heterogeneity was high in the subgroup with mild PD (χ^2^ = 11.28, *P* = 0.01; I^2^ = 73%) and moderate in the subgroup with severe PD (χ^2^ = 4.73, *P* = 0.09; I^2^ = 58%). The analysis of results stratified according to the security of PD diagnosis showed that a higher risk of PE was present in both subgroups (Figure 6), but it was significant only in the subgroup with insecure diagnosis (OR 2.68; 95% CI 1.64–4.37, *P*<0.0001). Heterogeneity was high in the subgroup with secure diagnosis (χ^2^ = 31.26, *P*<0.00001; I^2^ = 87%) and moderate in the subgroup with unsecure diagnosis (χ^2^ = 22.19, *P* = 0.008; I^2^ = 59%).

#### Publication bias

**Figure 7:**
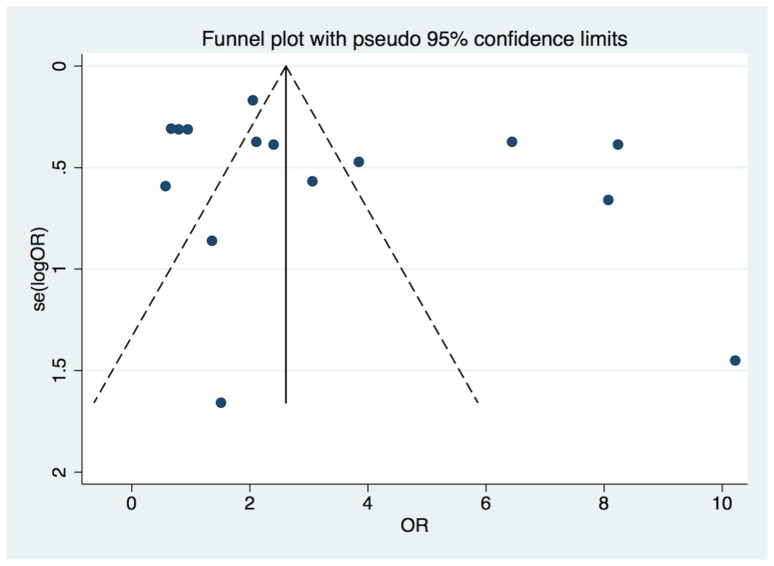
Funnel plot for the association between PD and PE.

**Figure 8:**
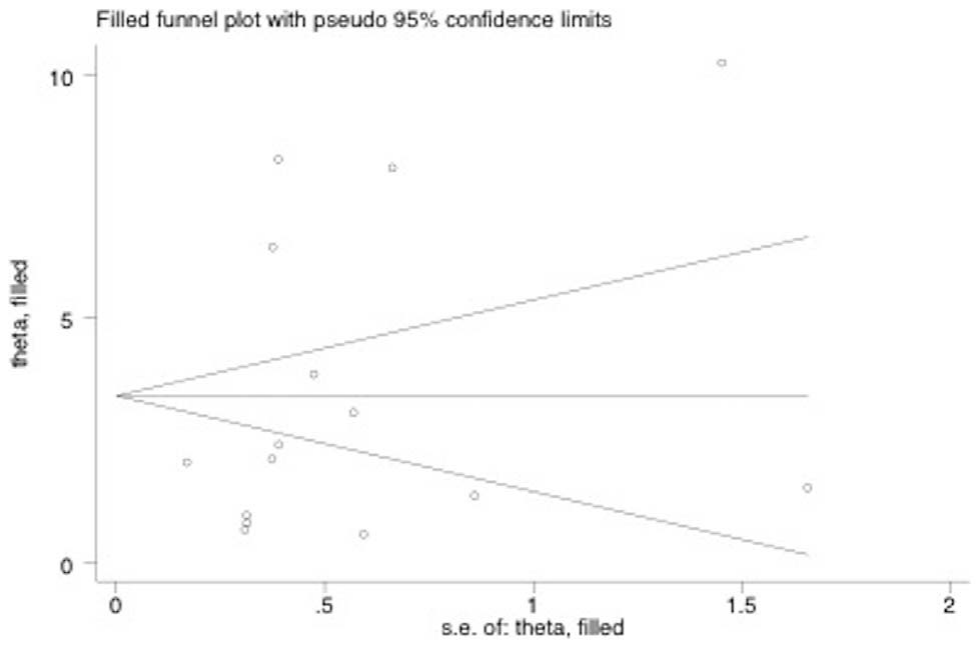
Trim and filled funnel plot for the association between PD and PE.

An inspection of the funnel plot seemed to reveal an asymmetry (Figure 7). However, the trim and fill analysis did not indicate any missing studies (OR 3.49, 95% CI, 2.24– 4.75, *P* = 0.2; Figure 8). Egger's regression asymmetry test indicated that the differences between the original estimate and the adjusted effect were not significant.

## Discussion

The aim of the present meta-analysis was to assess the potential association between PE and PD. The results seemed to indicate that PD is a risk factor for PE. Important differences were observed when the results were stratified into subgroups. In particular, when we analyzed the results according to the type of study design, the case-control studies, despite suffering from more confounding biases compared to cohort studies, revealed a significantly greater OR of PE for PD. The meta-analysis of cohort studies did not reveal any significant differences. However, the meta-analyses of subgroups potentially suffered from low power, due to the small number of included studies in each subgroup.

When analyzed according to the definition of PD, the meta-analysis performed with studies analyzing PD by PPD and CAL showed significant differences, whereas the meta-analysis of studies that defined PD by PPD alone or CAL alone did not. The definition of PD by PPD and CAL may be more appropriate, but only a few studies were included in the CAL and PPD subgroups (three and two studies, respectively). No differences were detected in the subgroup analysis of PD severity. Subgroup analysis for the security of PD diagnosis revealed that the insecure diagnosis subgroup, but not the secure diagnosis subgroup, reported a higher and significant risk of PE. Overall, given the small number of studies included in each subgroup and the corresponding low power to detect differences, it was difficult to assess the influence of the severity, the definition, and the security of diagnosis of PD on the association between PD and PE.

The present meta-analysis had several limitations. First, although meta-analysis is a useful tool in epidemiology, important issues related to methodology may limit its benefits. Among observational study designs, the case-control approach is not the best design. Thus, evidence from these studies is likely to be less accurate and possibly more influenced by recall bias compared to that from cohort studies. Second, we could not analyze the influence of the methodological quality on the results of the meta-analysis. Third, the funnel plot of publication bias was asymmetrical, and publication bias could not be excluded. This finding suggests that we may have missed important unpublished studies with results that are inconsistent with our findings. Nevertheless, the trim and fill analysis indicated that no other adjunctive study was missed. Egger's regression test revealed that the differences between the original and the adjusted analyses were not significant. The filled funnel plot showed that no additional unpublished study was needed. Fourth, although all of the included studies reported an adjusted analysis for important and known confounders, important differences were noted in the definitions of PD and PE. No general consensuses have been reached in the definition and diagnosis of PD [69]-[74]. The heterogeneity in these definitions may have influenced the results and introduced a bias into the meta-analysis. Therefore, given the methodological shortcomings, future studies are needed to confirm our results.

## Conclusions

Based on the findings of the meta-analysis, PD appears to be a possible risk factor for PE. However, the included studies demonstrated important differences in the definitions and diagnoses of PD and PE, and lacked good methodological quality. Therefore, future studies are needed to confirm the results of the present meta-analysis. These studies should have high methodological quality, with adjustment for known confounding factors, and should report a clear and secure diagnosis of PD.

## Supporting Information

Checklist S1 **PRISMA Checklist.**
(DOC)
